# The Changes and the Predictors of Suicidal Ideation Among HIV-positive Sexual Minority Men: A Five-year Longitudinal Study from China

**DOI:** 10.1007/s10461-021-03387-6

**Published:** 2021-07-31

**Authors:** Rui Luo, Vincent M. B. Silenzio, Yunxiang Huang, Xi Chen, Dan Luo

**Affiliations:** 1 Department of Social Medicine and Health Management, Xiangya School of Public Health, Central South University, 110 Xiangya Road, Changsha, Hunan 410078 People’s Republic of China; 2 Department of Urban-Global Public Health, Rutgers School of Public Health, Rutgers University, Newark, NJ USA; 3 Chinese Evidence-Based Medicine Center, West China Hospital, Sichuan University, Chengdu, Sichuan People’s Republic of China; 4 Hunan Provincial Center for Disease Prevention and Control, Changsha, Hunan People’s Republic of China

**Keywords:** Suicidal Ideation, Sexual Minority Men, HIV, Longitudinal Study

## Abstract

This study aimed to explore whether there were differences in suicidal ideation at different time points among sexual minority men (SMM) within five years of HIV diagnosis, and to investigate the influence of time and psychosocial variables on suicidal ideation. This was a five-year follow-up study focusing on the suicidal ideation among HIV-positive SMM who were recruited when they were newly diagnosed with HIV. Suicidal ideation and psychosocial characteristics including depression, anxiety, HIV-related stress, and social support were assessed within one month, the first year, and the fifth year after HIV diagnosis. A total of 197 SMM newly diagnosed with HIV completed three-time point surveys in this study. The prevalence of suicidal ideation was 27.4%, 15.7%, and 23.9% at one month, the first year, and the fifth year after HIV diagnosis, respectively. The risk of suicidal ideation was lower in the first year than baseline, but there was no significant difference between the fifth year and baseline. Emotional stress and objective support independently predicted suicidal ideation and they had interactions with time. The suicidal ideation of SMM newly diagnosed with HIV decreased in the first year and then increased in the fifth year, not showing a sustained decline trend in a longer trajectory of HIV diagnosis. Stress management, especially long-term stress assessment and management with a focus on emotional stress should be incorporated into HIV health care in an appropriate manner. In addition, social support should also be continuously provided to this vulnerable population.

**Supplementary Information:**

The online version contains supplementary material available at 10.1007/s10461-021-03387-6.

## Introduction

Suicidal behavior is recognized as a spectrum which covers a range of continuum acts from suicidal ideation, suicide plan, attempted suicide to completed suicide [1]. Suicidal ideation is the first step towards suicide and has been shown to predict later suicide plan, suicide attempt and completed suicide [2, 3]. In light of the key role of suicidal ideation in suicide, it has been recommended as an evaluation index for suicide prevention [4].

Suicide among sexual minority men (SMM) who are HIV-positive has become a growing major public health problem and has attracted concerns among scholars worldwide [5–9]. SMM are broadly defined (a) men who have sex with men, or (b) men who self-identify as gay or bisexual men, or (c) men who are intensely attracted to men [10]. Because of non-heterosexual orientation, SMM experience significant mental health problems in China, including suicidal ideation [11–14]. In recent years, the suicidal ideation of SMM has become a topic of increasing concern as the increasing HIV prevalence among SMM [15]. A meta-analysis in 2017 pointed out that the pooled lifetime prevalence of suicidal ideation was 34.97% among general SMM [16]. In addition, a few published studies have concluded that HIV-positive SMM are more likely to experience suicidal ideation [5–9]. Therefore, this vulnerable subgroup deserves more research attention.

Suicidal ideations are affected by some common psychosocial factors, including HIV-related stress, depression, anxiety, and social support. HIV-related stress refers to stressfulness from HIV/AIDS-specific stressors and can take various forms, such as concerns about disclosure, hesitation of antiretroviral therapy (ART), HIV-related stigma, and worry about physical changes [17]. Studies have shown that HIV-related stress was associated with worse mental health and even suicidal ideation [18, 19]. Depression and anxiety are two common types of emotional distress that have also been shown to be risk factors for suicidal ideation among HIV-positive SMM [20, 21]. In addition, social support has been widely recognized as a moderator that can buffer the negative effects of HIV diagnosis among SMM, including suicidal ideation [22]. It is thus important to understand these psychosocial variables to identify the risk of suicidal ideation and implement further targeted intervention to reduce such risk among HIV-positive SMM.

Suicidal ideation is also a variable that may change overtime, particularly in the first year after HIV diagnosis [23]. There may be disparities in suicidal ideation at different time points after HIV diagnosis. But to our knowledge, most studies on suicidal ideation and associated factors among SMM living with HIV are cross-sectional [5–9]. There is a lack of longitudinal study, especially for longer than one-year follow-up study. In addition, some psychosocial characteristics, such as social support and stress, are also time-dependent variables [24]. It is thus important to understand how the effects of psychosocial characteristics on suicidal ideation change over time. The current study was conducted to fill in the research gap with the following purposes: (1) to explore whether there were differences in suicidal ideation among SMM with newly diagnosed HIV at different time points within five years. (2) to investigate the psychosocial factors associated with suicidal ideation among SMM living with HIV after adjusting for the covariates.

## Methods

### Participants

This five-year longitudinal observational study was conducted at the HIV Voluntary Counselling and Testing (VCT) outpatient department of Center for Disease Control and Prevention (CDC) in Changsha, Hunan Province, China. Participants were consecutively recruited from 1st March 2013 to 30th September 2014. Eligible participants had to meet the following inclusion criteria: (a) aged above 18 years old, (b) resided in Changsha over 6 months, (c) diagnosed as HIV infection for no more than one month.

### Setting and Procedure

We established a cohort of individuals who had been newly diagnosed with HIV. Baseline survey was conducted between 1st March 2013, and 30th September 2014 at Changsha CDC. The first and second follow-up surveys were conducted at the Changsha CDC and Changsha Hospital for Infectious Diseases in the first year and the fifth year after HIV diagnosis, respectively. For the two follow-up surveys, information on participants who had and had not initiated ART was collected at the Changsha Hospital for Infectious Diseases, and Changsha CDC, respectively. After providing written informed consent, all participants were invited to complete a questionnaire by face-to-face interviews at baseline and two follow-up time points. With the consent of the participants, our team used the Chinese HIV/AIDS Comprehensive Response Information Management System (CRIMS) to obtain HIV-related clinical information, including whether the participants had initiated antiretroviral therapy (ART) during the two follow-up periods and their CD4 cell counts.

### Measurements

#### Dependent Variable

##### Suicidal Ideation

In this study, the main outcome suicidal ideation was assessed by two questions based on two items adapted from the section of World Mental Health-Composite International Diagnostic Interview (WMH-CIDI) suicidality assessment [25], which were also used in other similar studies [26–28]. In the baseline survey, participants were asked “Have you seriously considered taking suicide after HIV diagnosis?” and in the two follow-up surveys, participants were asked "Have you seriously considered taking suicide in the last year?" The answer of each question is dichotomized, with “yes” representing presence of suicidal ideation.

#### Explanatory Variables

##### Depressive Symptoms

Depressive symptoms were assessed by the 9-item Patient Health Questionnaire Depression Scale (PHQ-9), which is a 4-point Likert-type scale ranging from 0 to 3 [29]. A higher score indicates more severe depressive symptoms, with a cut-off point of 10 for significant depressive symptoms [30]. In this study, we used the Chinese version of PHQ-9 translated by Wang et al., which has shown good reliability and validity [31]. In the current study, the PHQ-9 showed good internal consistency with a Cronbach's α coefficient of 0.903.

##### Anxiety Symptoms

Anxiety symptoms were assessed by the 7-item Generalized Anxiety Disorder Questionnaire (GAD-7), which is a 4-point Likert-type scale ranging from 0 to 3 [32]. A higher score indicates more severe anxiety symptoms, with a cur-off point of 10 for significant anxiety symptoms [33]. In this study, we used the Chinese version of GAD-7 translated by He et al., which has shown good reliability and validity [34]. In the current study, the GAD-7 showed good internal consistency with a Cronbach's α coefficient of 0.936.

##### HIV-Related Stress

HIV-related stress was measured by the 17-item Chinese HIV/AIDS Stress Scale (CSS-HIV), which covers three dimensions: emotional stress, social stress, and instrumental stress. It is a 5-point Likert-type scale, with a higher score suggesting a higher stress level [35]. The scale was originally compiled by Pakenham [36] and later translated into Chinese by Niu et al., which has shown good reliability and validity [35]. In the current study, the CSS-HIV showed good internal consistency with a Cronbach's α coefficient of 0.911.

##### Social Support

Social support was assessed by the 10-item Social Support Rating Scale (SSRS), which covers three dimensions: objective support, subjective support and support utilization [37]. The total score ranges from 12 to 66, with a higher score indicating more social support. In this study the SSRS showed good internal consistency with a Cronbach’s α coefficient of 0.820.

#### Covariates

##### Socio-Demographic Information

Socio-demographic information was collected by a questionnaire, which included: age (18–29, > 29), marital status (married, unmarried), sexual orientation (gay, bisexual), monthly income (≤ 4000, > 4000 yuan), education (college or higher, senior or lower), employment (employed, unemployed), household registration (rural, urban).

##### HIV-Related Clinical Information

HIV-related clinical information was collected from CRIMS, which included CD4 counts and antiretroviral therapy (ART) initiation status.

### Statistical Analysis

Descriptive statistics were expressed as the median of frequency, percentage, and interquartile range (IQR). In order to compare the baseline sample characteristics of participants who completed the follow-up surveys and those who dropped out, the chi-square test was used for comparison of categorical variables, and the Mann–Whitney U test was used for comparison of continuous variables.

We used the logistic regression with generalized estimation equation (GEE) method to determine time and psychosocial factors (depressive and anxiety symptoms, HIV-related stress, and social support) associated with suicidal ideation among SMM newly diagnosed with HIV. One advantage of the GEE method is the applicability of a wide range of data to dependent variables for repeated measurements [38]. With suicidal ideation as the dependent variable, 3 independent multivariable GEE models were used for the study. In Model 1, we entered only time factors and depressive and anxiety symptoms. In Model 2, we added three dimensions of HIV-related stress scores based on Model 1. In Model 3, we included three dimensions of social support scores based on model 2. To see whether the effects of psychosocial variables on suicidal ideation changed over time, we examined the interaction between time factors and significant psychosocial variables in multivariate GEE models. All models adjusted for age, marital status, education, household registration, sexual orientation, employment, monthly income, CD4 cell counts, and ART initiation status. P < 0.05 was considered statistically significant. All data analyses were performed using SPSS for Windows 26.0 (SPSS, Inc., Chicago, IL, USA).

### Ethical Approval and Consideration

All participants provided written informed consent. In addition, an emergency plan was developed for participants who presented serious suicidal ideation during the interview. A reporting process will be initiated according to the emergency plan, and the investigator immediately informed the CDC staff, who would take appropriate procedures to help the participants. Moreover, the study team will also provide referral information of professional psychological crisis intervention institutions for participants in need.

## Results

### Sample Characteristics

**Fig. 1:**
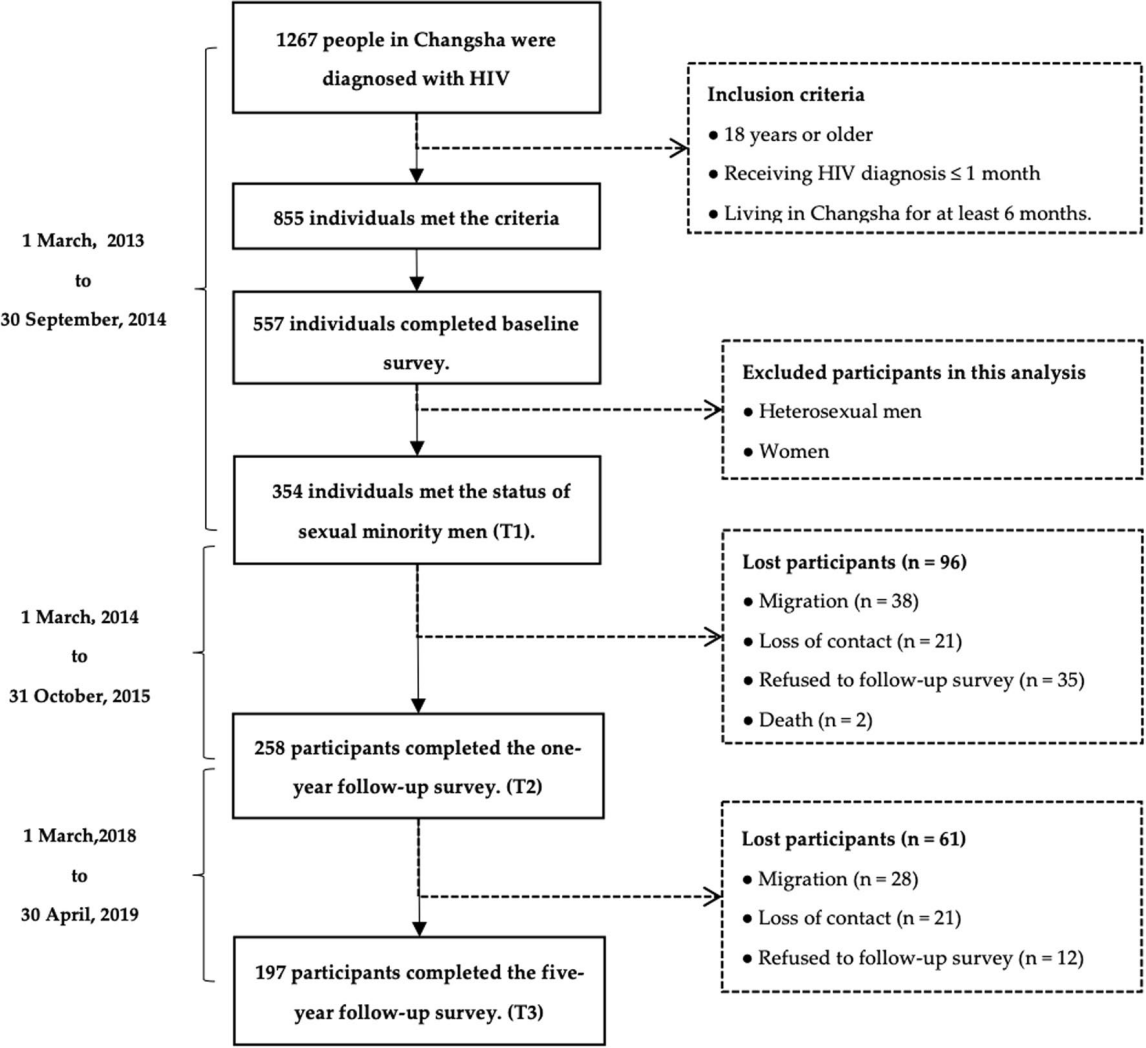
Flowchart of participant enrollment

A total of 1,267 people was newly diagnosed with HIV in Changsha during the baseline survey period. Of the 855 people who met the criteria for inclusion, 557 participated in the study. We finally included a total of 354 individuals who reported themselves as SMM in this study, 258 of whom completed the first follow-up survey and 197 completed the second follow-up survey. Figure 1 shows the detailed flowchart of participant enrollment.

All baseline sample characteristics were not significantly different between 197 participants who completed the three-time surveys and 157 of those lost to follow-up (Supplementary Table S1).

Table 1 shows baseline sample characteristics. Most of the participants who completed the second follow-up survey were in the 18–29 age group (65.5%), unmarried (84.8%), gay (65.0%), employed (71.1%).

**Table 1:** Sample characteristics at baseline

Characteristics	N (%)
Age	
18–29	129 (65.5%)
> 29	68 (34.5%)
Marital status	
Married	30 (15.2%)
Unmarried	167 (84.8%)
Sexual orientation	
Gay	128 (65.0%)
Bisexual	69 (35.0%)
Household registration	
Rural	88 (44.7%)
Urban	109 (55.3%)
Education	
Senior or lower	89 (45.2%)
College or higher	108 (54.8%)
Employment	
Employed	140 (71.1%)
Unemployed	57 (28.9%)
Monthly income (RMB)	
≤ 4000	113 (57.3%)
> 4000	84 (42.7%)
CD4 count, cells/mm3	
≤ 350	83 (42.1%)
> 350	114 (57.9%)

### The Trajectory of Suicidal Ideation and Psychological Characteristics

**Fig. 2:**
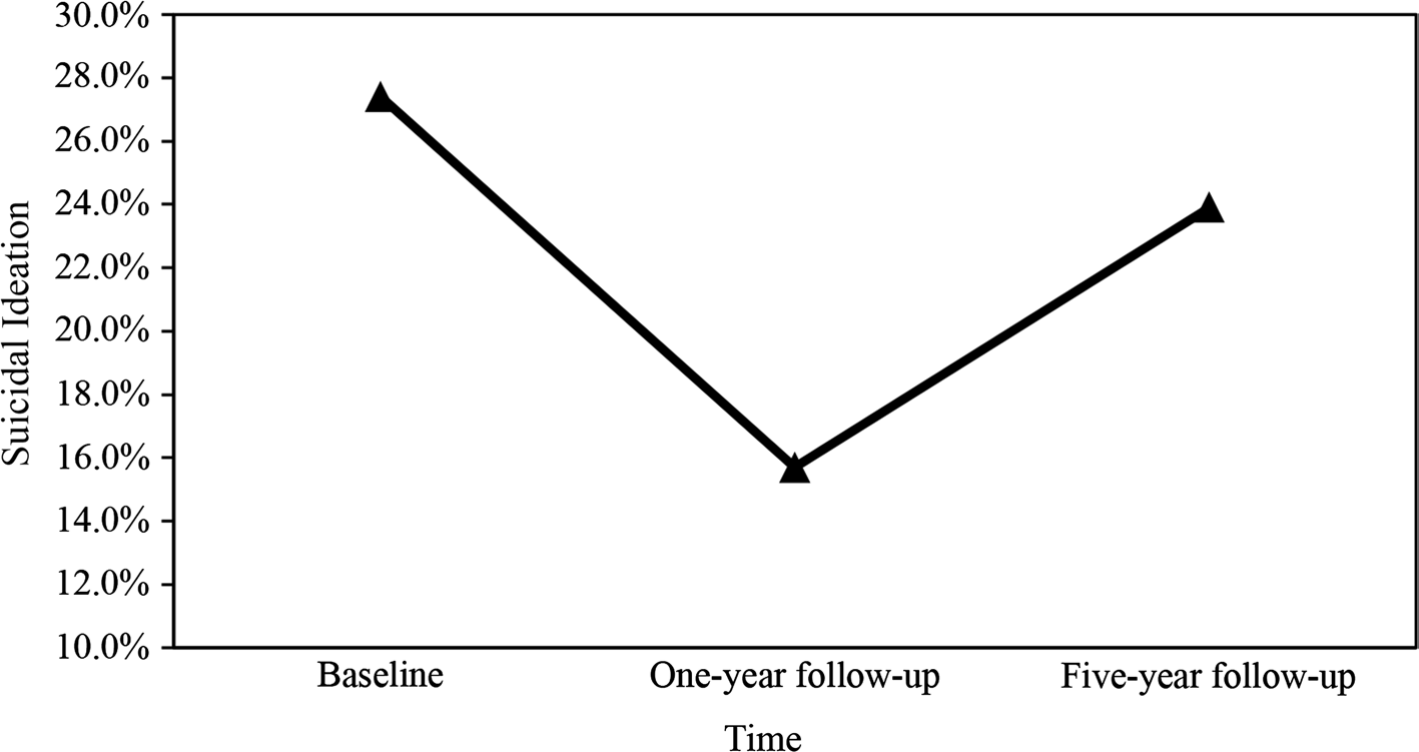
Longitudinal trajectory of suicidal ideation

Table 2 shows the differences in suicidal ideation and psychosocial characteristics between the baseline and two follow-up surveys. The prevalence of suicidal ideation was 27.4% at baseline, 15.7% after one year, and 23.9% after five years. In terms of psychosocial characteristics, the prevalence of depressive symptoms was 42.1% at baseline, 12.2% after one year, and 16.2% after five years. The prevalence of anxiety symptoms was 29.4% at baseline, 12.7% after one year, and 11.2% after five years. In addition, the five-year trajectory of suicidal ideation among the HIV-positive SMM is illustrated in Fig. 2.

**Table 2:** Description in suicidal ideation and psychosocial characteristics at three-time points

Characteristics	Baseline	One-year follow-up	five-year follow-up
Suicidal ideation			
Yes	54 (27.4%)	31 (15.7%)	47 (23.9%)
No	143 (72.6%)	166 (84.3%)	150 (76.1%)
Depressive symptoms			
No significant	114 (57.9%)	173 (87.8%)	165 (83.8%)
Significant	83 (42.1%)	24 (12.2%)	32 (16.2%)
Anxiety symptoms			
No significant	139 (70.6%)	172 (87.3%)	175 (88.8%)
Significant	58 (29.4%)	25 (12.7%)	22 (11.2%)
HIV-related stress, median (IQR)			
Emotional stress	5 (3, 10)	3 (1, 6)	3 (0, 5)
Social stress	11 (7, 16)	7 (4, 11)	8 (5, 13)
Instrumental stress	4 (1, 6)	2 (0, 5)	2 (0, 5)
Social support, median (IQR)			
Subjective support	13 (10, 17)	13 (10, 19)	18 (15, 21)
Objective support	8 (6, 10)	6 (4, 8)	6 (4, 7)
Support utilization	6 (5, 7)	6 (5, 7)	6 (5, 7)

### Suicidal Ideation at Each Time Point

Table 3 shows the proportion of participants with or without suicidal ideation at each time point. 51.7% of participants did not report suicidal ideation at all three time points. 13.7% of participants reported suicidal ideation only at baseline. 12.2% of participants reported suicidal ideation only in the fifth year. In addition, 3.6% of participants reported suicidal ideation at all three time points.

**Table3:** Suicidal ideation at each point time

Suicidal ideation			N (%)
Baseline	One-year follow-up	Five-year follow-up	
Yes	Yes	Yes	7 (3.6%)
Yes	Yes	No	9 (4.6%)
Yes	No	No	27 (13.7%)
Yes	No	Yes	11 (5.6%)
No	Yes	Yes	5 (2.5%)
No	No	Yes	24 (12.2%)
No	Yes	No	12 (6.1%)
No	No	No	102 (51.7%)

### Factors Associated With Suicidal Ideation

Table 4 shows risk factors associated with suicidal ideation among SMM newly diagnosed with HIV at the three-time points using multivariate GEE models. Associated factors of suicidal ideation at three-time points included in model 3: first year after HIV diagnosis (OR: 0.35; 95% CI: 0.17, 0.70); unmarried (OR: 0.41; 95% CI: 0.22, 0.75), bisexuality (OR: 1.87; 95% CI: 1.12, 3.11), ART initiation (OR: 2.55; 95% CI: 1.20, 5.43), emotional stress (OR: 1.23; 95% CI: 1.13, 1.33), and objective support (OR: 0.86; 95% CI: 0.77, 0.96).

**Table 4:** Factors associated with suicidal ideation among SMM living with HIV

Characteristics	Model 1 OR (95% CI)	*p*-value	Model 2 OR (95% CI)	*p*-value	Model 3 OR (95% CI)	*p*-value
Time						
Baseline (T1)	Ref		Ref		Ref	
One-year follow-up (T2)	0.39 (0.21, 0.73)	0.003	0.45 (0.24, 0.86)	0.015	0.35 (0.17, 0.70)	0.003
Five-year follow-up (T3)	0.48 (0.20, 1.19)	0.113	0.66 (0.26, 1.65)	0.370	0.46 (0.17, 1.25)	0.128
Age						
18–29	Ref	0.593	Ref	0.687	Ref	0.301
> 30	0.88 (0.55, 1.41)		0.91 (0.56, 1.47)		0.78 (0.48, 1.25)	
Marital status						
Married	Ref	0.004	Ref	0.007	Ref	0.004
Unmarried	0.47 (0.28, 0.78)		0.47 (0.27, 0.82)		0.41 (0.22, 0.75)	
Sexual orientation						
Gay	Ref	0.079	Ref	0.028	Ref	0.016
Bisexual	1.53 (0.95, 2.47)		1.74 (1.06, 2.87)		1.87 (1.12, 3.11)	
Household registration						
Rural	Ref	0.753	Ref	0.449	Ref	0.374
Urban	0.93 (0.58, 1.49)		0.82 (0.50, 1.37)		0.79 (0.48, 1.32)	
Education						
Senior or lower	Ref	0.448	Ref	0.301	Ref	0.355
College or higher	1.21 (0.74, 1.99)		1.32 (0.78, 2.23)		1.29 (0.75, 2.22)	
Employment						
Employed	Ref	0.176	Ref	0.356	Ref	0.488
Unemployed	1.42 (0.86, 2.34)		1.27 (0.77, 2.10)		1.22 (0.70, 2.11)	
Monthly income (RMB)						
≤ 4000	Ref	0.607	Ref	0.716	Ref	0.715
> 4000	0.89 (0.56, 1.40)		0.92 (0.58, 1.46)		0.91 (0.55, 1.50)	
CD4 count, cells/mm3						
≤ 350	Ref	0.551	Ref	0.741	Ref	0.764
> 350	1.16 (0.71, 1.88)		1.09 (0.66, 1.78)		1.08 (0.69, 1.81)	
ART initiation status						
No	Ref	0.020	Ref	0.033	Ref	0.015
Yes	2.43 (1.15, 5.13)		2.32 (1.07, 5.05)		2.55 (1.20, 5.43)	
Depressive symptoms						
No significant	Ref	0.026	Ref	0.979	Ref	0.972
Significant	1.98 (1.09, 3.60)		1.01 (0.48, 2.14)		0.99 (0.44, 2.10)	
Anxiety symptoms						
No significant	Ref	0.154	Ref	0.945	Ref	0.731
Significant	1.60 (0.84, 3.06)		0.98 (0.48, 1.97)		0.87 (0.41, 1.88)	
HIV-related stress						
Emotional stress	N/A	N/A	1.25 (1.15, 1.36)	< 0.001	1.23 (1.13, 1.33)	< 0.001
Social stress	N/A	N/A	0.96 (0.91, 1.01)	0.112	0.96 (0.91, 1.01)	0.145
Instrumental stress	N/A	N/A	0.96 (0.88, 1.05)	0.376	0.98 (0.89, 1.07)	0.992
Social support						
Subjective support	N/A	N/A	N/A	N/A	0.99 (0.95, 1.04)	0.777
Objective support	N/A	N/A	N/A	N/A	0.86 (0.77, 0.96)	0.006
Support utilization	N/A	N/A	N/A	N/A	1.04 (0.90, 1.21)	0.580

^*^Model (1): Sociodemographic characteristics + HIV-related clinical information + Time + Depressive and anxiety symptoms

Model (2): Sociodemographic characteristics + HIV-related clinical information + Time + Depressive and anxiety symptoms + HIV-related stress

Model (3): Sociodemographic characteristics + HIV-related clinical information + Time + Depressive and anxiety symptoms + HIV-related stress + Social support

### Interactions Between Psychosocial Variables and Time

Table 5 shows interaction effect between time and psychosocial variables at three time points by multivariate analysis. Significant negative interaction between time and emotional stress, and significant positive interaction between time and objective support were found at three time points.

**Table 5:** The interaction of time with significant psychosocial variables

Variable	uOR (95% CI)	*p*-value	aOR (95% CI)	*p*-value
Model 1				
Time				
Baseline (T1)	Ref		Ref	
One-year follow-up (T2)	1.75 (0.95, 3.21)	0.071	1.83 (0.95, 3.52)	0.071
Five-year follow-up (T3)	4.94 (2.21, 11.01)	< 0.001	5.82 (2.28, 14.85)	< 0.001
Emotional stress	1.45 (1.30, 1.62)	< 0.001	1.51 (1.31, 1.74)	< 0.001
Time × Emotional stress	0.91 (0.86, 0.95)	< 0.001	0.89 (0.84, 0.95)	< 0.001
Model 2				
Time				
Baseline (T1)	Ref		Ref	
One-year Follow-up (T2)	0.15 (0.07, 0.31)	< 0.001	0.22 (0.05, 0.91)	< 0.001
Five-year follow-up (T3)	0.10 (0.03, 0.29)	< 0.001	0.18 (0.05, 0.63)	0.006
Objective support	0.63 (0.53, 0.72)	< 0.001	0.67 (0.55, 0.82)	< 0.001
Time × Objective support	1.16 (1.07, 1.26)	< 0.001	1.13 (1.04, 1.23)	0.005

^*^Models adjust for age, marital status, household registration, education, employment, Monthly income, sexual orientation, CD4 counts and ART initiation status

## Discussion

In this study, the baseline prevalence of suicidal ideation among SMM when they were newly diagnosed with HIV was 27.4%, which was higher than the lifetime prevalence of suicidal ideation among the general Chinese SMM [39]. The risk of suicidal ideation was lower one year after HIV diagnosis than one month within HIV diagnosis. However, there was no significant difference in the risk of suicidal ideation between the fifth year after HIV diagnosis and one month within HIV diagnosis. Emotional stress and objective support were independent predictors for suicidal ideation. There are interactions between time and emotional stress and objective support during the five-year follow-up period. The effect of emotional stress on suicidal ideation had slightly diminished over time, while the effect of objective support on suicidal ideation had slightly increased over time. Other factors, including married status and ART initiation status were also shown to be risk factors for suicidal ideation in both type of models, which may provide some new viewpoints for suicide prevention among SMM living with HIV.

Our data revealed that risk of suicidal ideation had decreased over time during the first year after diagnosis. This may be explained by the phenomenon of post-traumatic growth which refers to the positive psychological consequences of struggling with a traumatic event [40]. Some studies have found that the pathogenic effects of trauma are more common among people living with HIV than general population, even in a society with universal access to effective HIV-related medical health care [41, 42]. With the widespread use of ART, HIV infection has transformed from an acute life-threatening disease into a chronic remediable disease that can be managed. As Rzeszutek revealed in a systematical review, although being diagnosed with HIV is a traumatic event that can be stressful, many people living with HIV have positively learned HIV-related knowledge, actively sought for medical care, and gradually recognized that HIV infection was treatable [43]. Thus, they have adapted to the HIV-positive status over time and gained a new commitment to personal goals and life [44].

Another important finding was that the risk of suicidal ideation was not significantly different in the fifth year after HIV diagnosis from one month within HIV diagnosis. Contrary to our expectation, there was no sustained declining or flat trend in suicidal ideation in the longer trajectory of HIV infection over five years. This finding may be related to HIV-related stigma. During the post-traumatic period, additional distress caused by HIV-related stigma was negatively associated with post-traumatic growth outcomes [45]. In China, people living with HIV are mostly infected through activities that are usually considered immoral, especially for SMM who are considered promiscuous [46, 47]. Moreover, in traditional Chinese culture, SMM themselves are stigmatized due to their sexual minority status [48]. When SMM are infected, they will gradually face more troubles in life, work and interpersonal relationships than general people living with HIV because of the “double stigma” [49]. Furthermore, HIV-positive SMM face many difficulties in finding a same-sex sexual partner if their HIV-positive status is known to others [50]. Therefore, HIV clinicians should recognize that suicide ideation not only peak soon after HIV diagnosis, but also increased after a longer period of HIV diagnosis among SMM. This finding has implications for future suicide prevention program to screen for suicidal ideation at multiple stages after the diagnosis of HIV infection among SMM.

We found that the emotional stress dimension of HIV-related stress was associated with suicidal ideation. When emotional stress scores were higher, the risk of suicidal ideation was higher. In China, personal motivation (such as feeling depressed, desperate and wanting to escape pain) was more recognized as risk factors of suicidal ideation than interpersonal factors [51, 52]. In addition, according to the minority stress theory, SMM is a social subgroup that is vulnerable to stigma and discrimination due to same-sex behavior or orientation, which makes them more prone to excessive stress and mental health disorders [53]. SMM living with HIV often face “double stress” from not only HIV infection, but also sexual minority status [53]. Moreover, the interaction results at three-time point showed that the effect of emotional stress on suicidal ideation diminished slightly over time during a five-year period after HIV infection. This finding indicates there is a strong need to integrate mental health services especially stress management within HIV care facilities for SMM living with HIV in an appropriate manner.

Depression and anxiety are recognized as important risk factors for suicidal ideation [54, 55]. The presence of a clinically active major depressive episode may be a strong predictor of suicidal ideation [56]. In GEE Model 1, we found that depressive and anxiety symptoms are risk factors for suicidal ideation, which is consistent with most studies [57, 58]. However, this effect disappeared when HIV-related stress was included in the GEE model. The findings suggest that HIV-related stress is a stronger predictor of suicidal ideation than depression and anxiety symptoms among SMM living with HIV. This may be due to the fact that individuals may have a transient increase in emotional response and develop transient, sudden suicidal ideation following excessive stress [59, 60]. These findings suggest that psychosocial characteristics are important predictors of suicide ideation. There is an urgent need to increase psychological counseling services for common psychological disorders such as depression, anxiety symptoms, and stress in HIV management and care.

This study found that higher objective support dimension of social support scores were associated with lower risk of suicidal ideation. This suggests that practical support, such as direct financial assistance, social networks of family, friends, and colleagues, as well as the presence and involvement of group relationships, can persistently help SMM to cope better with the stress of being diagnosed with HIV. Unfortunately, the overall level of social support in the participants is low. Moreover, the interaction results showed that the effect of objective support on suicidal ideation enhanced slightly over time during the five-year period after HIV infection. This finding suggest that social support need to be provided continuously to SMM living with HIV. One thing noteworthy is that the risk of suicidal ideation was higher in the married participants, contrary to previous studies in general people living with HIV [61]. In China, most of SMM choose to hide their real sexual orientation and marry women under stress from society and family [62]. According to a previous study, about 80% of SMM eventually married a woman in China [63]. After being diagnosed with HIV, SMM not only face the psychological stress of disclosing their HIV status to their wives, but also the additional stress of disclosing their sexual minority. Therefore, it is important to recognize that marriage does not increase the level of social support for Chinese SMM living with HIV.

Our study showed that participants who initiated ART had a higher risk of suicidal ideation, which is contrary to previous study [64]. This finding may be partly explained by the side effects of ART. For instance, efavirenz containing non-nucleoside reverse transcriptase inhibitor (NNRTI) in ART may cause severe adverse reactions in the central nervous system [65]. The drug can cause mental disorders such as severe depression and suicidal ideation. In addition, people who have just initiated ART may fear that their HIV-positive status will be exposed, which constitutes a huge barrier for drug adherence [66]. Therefore, maladaptive drug side effects and poor drug adherence may contribute to increased suicidal ideation during the early stage of ART treatment ^67^. This finding suggests that special attention need to be paid to the mental health status of people infected with HIV who just initiated ART, and during the long-term treatment process.

There are limitations to this study. First, the non-random sampling method may limit the generalization of the results in this study. Second, we rely on self-reported scales to assess psychosocial characteristic. In order to provide a more reliable measure of psychosocial characteristics, professional diagnostic tools should be included in the assessment of psychosocial characteristics in future studies. Third, the time intervals of our longitudinal data collection are unbalanced, this may cause some information to lose during the five-year follow-up periods. Finally, more than 40% of the participants dropped out at five-year follow-up, which may bring about potential bias. However, a careful comparison of sample characteristics between those who dropped out and those who retained showed no significant difference, indicating that our conclusion should be valid (Details are provided in Table S1).

## Conclusion

The suicidal ideation of SMM living with HIV decreased in the first year and then increased in the fifth year, not showing a sustained decline trend in a longer trajectory of HIV diagnosis. Our findings may have considerable implications for HIV clinicians and relevant policy makers to develop more effective interventions to reduce the risk of suicidal ideation among SMM living with HIV not only in the early stages of HIV diagnosis, but also in the longer period after HIV diagnosis. Moreover, we need to provide timely professional psychological crisis intervention when suicidal ideation is found. Stress management, especially long-term stress assessment and management with a focus on emotional stress should be incorporated into HIV health care in an appropriate manner. In addition, social support should also be continuously provided to this vulnerable population.

## Supplementary Information

Below is the link to the electronic supplementary material.

Supplementary file1 (docx 18 kb)

## Data Availability

The data sets used and analysed in the study are available from the corresponding author on reasonable request.
