# Supplementary material for: The Changes and the Predictors of Suicidal Ideation Among HIV-positive Sexual Minority Men: A Five-year Longitudinal Study from China
Source: AIDS Behav. 2021 Jul 31;26(2):339–49. doi: 10.1007/s10461-021-03387-6 (PMC8813835; doi:10.1007/s10461-021-03387-6)
Supplement: Supplementary file 1 — Supplementary file1 (docx 18 kb) [file 10461_2021_3387_MOESM1_ESM.docx]

Table S1 Sample characteristics between retained and dropout clients at 5- year follow-up

| Characteristics | Baseline Total (n=354) | Retained  (n=197) | Dropped out (n=157) | Test ($\mathrm{Chi}^{2}$/Z) | p-value |
| --- | --- | --- | --- | --- | --- |
| Age |  |  |  |  |  |
| 18-29 | 238 (67.2%) | 129 (65.5%) | 109 (69.4%) | 0.617 | 0.432 |
| >29 | 116 (32.8%) | 68 (34.5%) | 48 (30.6%) |  |  |
| Marital status |  |  |  |  |  |
| married | 51 (14.4%) | 30 (15.2%) | 21 (13.4%) | 0.243 | 0.622 |
| unmarried | 303 (85.6%) | 167 (84.8%) | 136 (86.6%) |  |  |
| Sexual orientation |  |  |  |  |  |
| Gay | 235 (66.4%) | 128 (65.0%) | 107 (68.2%) | 0.395 | 0.529 |
| Bisexual | 119 (33.6%) | 69 (35.0%) | 50 (31.8%) |  |  |
| Household registration |  |  |  |  |  |
| Rural | 162 (45.8%) | 88 (44.7%) | 74 (47.1%) | 0.214 | 0.644 |
| Urban | 192 (54.2%) | 109 (55.3%) | 83 (52.9%) |  |  |
| Education |  |  |  |  |  |
| Senior or lower | 151 (42.7%) | 89 (45.2%) | 63 (40.1%) | 0.910 | 0.340 |
| College or higher | 203 (57.3%) | 108 (54.8%) | 94 (59.9%) |  |  |
| Employment |  |  |  |  |  |
| Employed | 253 (71.5%) | 140 (71.1%) | 113 (72.0%) | 0.035 | 0.851 |
| Unemployed | 101 (28.5%) | 57 (28.9%) | 44 (28.0%) |  |  |
| Monthly income (RMB) |  |  |  |  |  |
| ≤ 4000 | 202 (57.1%) | 113 (57.3%) | 90 (57.3%) | <0.001 | 0.995 |
| >4000 | 152 (42.9%) | 84 (42.7%) | 67 (42.7%) |  |  |
| CD4 count, cells/mm3 |  |  |  |  |  |
| ≤ 350 | 148 (41.8%) | 83 (42.1%) | 58 (36.9%) | 0.982 | 0.322 |
| > 350 | 206 (58.2%) | 114 (57.9%) | 99 (63.1%) |  |  |
| Suicidal ideation |  |  |  |  |  |
| Yes | 99 (28.0%) | 54 (27.4%) | 45 (28.7%) | 0.068 | 0.794 |
| No | 255 (72.0%) | 143 (72.6%) | 112 (71.3%) |  |  |
| Depressive symptoms |  |  |  |  |  |
| No significant | 208 (58.8%) | 114 (57.9%) | 95 (60.5%) | 0252 | 0.616 |
| significant | 146 (41.2%) | 83 (42.1%) | 62 (39.5%) |  |  |
| Anxiety symptoms |  |  |  |  |  |
| No significant | 245 (69.2%) | 139 (70.6%) | 107 (68.2%) | 0.238 | 0.625 |
| significant | 109 (30.8%) | 58 (29.4%) | 50 (31.8%) |  |  |
| HIV-related stress, median (IQR) |  |  |  |  |  |
| Emotional stress | 6 (3, 10) | 5 (3, 10) | 6 (3, 10) | -0.531 | 0.595 |
| Social stress | 12 (7, 16) | 11 (7, 16) | 13 (3, 10) | -1.325 | 0.185 |
| Instrumental stress | 4 (1, 7) | 4 (1, 6) | 4 (2, 8) | -1.269 | 0.204 |
| Social support, median (IQR) |  |  |  |  |  |
| Subjective support | 14 (10, 18) | 13 (10, 17) | 14 (11, 19) | -1.405 | 0.160 |
| Objective support | 8 (6, 9) | 8 (6, 10) | 8 (5, 9) | 0.908 | 0.364 |
| Support utilization | 6 (5, 7) | 6 (5, 7) | 6 (5, 8) | 0.059 | 0.953 |
